# OsPhyB-Mediating Novel Regulatory Pathway for Drought Tolerance in Rice Root Identified by a Global RNA-Seq Transcriptome Analysis of Rice Genes in Response to Water Deficiencies

**DOI:** 10.3389/fpls.2017.00580

**Published:** 2017-04-26

**Authors:** Yo-Han Yoo, Anil K. Nalini Chandran, Jong-Chan Park, Yun-Shil Gho, Sang-Won Lee, Gynheung An, Ki-Hong Jung

**Affiliations:** Graduate School of Biotechnology & Crop Biotech Institute, Kyung Hee UniversityYongin, South Korea

**Keywords:** *phyb* mutant, rice (*Oryza sativa*), RNA-seq, root, water deficiency

## Abstract

Water deficiencies are one of the most serious challenges to crop productivity. To improve our understanding of soil moisture stress, we performed RNA-Seq analysis using roots from 4-week-old rice seedlings grown in soil that had been subjected to drought conditions for 2–3 d. In all, 1,098 genes were up-regulated in response to soil moisture stress for 3 d, which causes severe damage in root development after recovery, unlikely that of 2 d. Comparison with previous transcriptome data produced in drought condition indicated that more than 68% of our candidate genes were not previously identified, emphasizing the novelty of our transcriptome analysis for drought response in soil condition. We then validated the expression patterns of two candidate genes using a promoter-GUS reporter system in planta and monitored the stress response with novel molecular markers. An integrating omics tool, MapMan analysis, indicated that RING box E3 ligases in the ubiquitin-proteasome pathways are significantly stimulated by induced drought. We also analyzed the functions of 66 candidate genes that have been functionally investigated previously, suggesting the primary roles of our candidate genes in resistance or tolerance relating traits including drought tolerance (29 genes) through literature searches besides diverse regulatory roles of our candidate genes for morphological traits (15 genes) or physiological traits (22 genes). Of these, we used a T-DNA insertional mutant of *rice phytochrome B (OsPhyB)* that negatively regulates a plant's degree of tolerance to water deficiencies through the control of total leaf area and stomatal density based on previous finding. Unlike previous result, we found that *OsPhyB* represses the activity of ascorbate peroxidase and catalase mediating reactive oxygen species (ROS) processing machinery required for drought tolerance of roots in soil condition, suggesting the potential significance of remaining uncharacterized candidate genes for manipulating drought tolerance in rice.

## Introduction

Water deficiencies at critical growth stages can seriously restrict crop productivity. One of the greatest challenges in providing adequate soil moisture is the erratic patterns and reduced amounts of rainfall due to climate change. Severe droughts now occur almost every year in rain-fed rice-growing regions of the world, drastically affecting yields from more than 20 million ha in South and Southeast Asia and nearly 80% of the area planted to rice in Africa (Pandey et al., 2007). Drought conditions can influence productivity to varying degrees depending upon the time of onset, duration, and intensity. For example, when plants are stressed during their reproductive stage, average yield losses can be more than 50% (Boyer, 1982; Venuprasad et al., 2007).

Especially, functional identification of novel candidate genes will be valuable for future applications using gene editing or overexpression analyses. In rice, <3% of non-transposable element genes (1,022) in rice have been functionally characterized (Chandran et al., 2016). Information on the functionally characterized genes in rice is well-summarized in the Overview of functionally characterized Genes in Rice Online database (OGRO) on the Q-TARO website (http://qtaro.abr.affrc.go.jp/ogro). Regarding drought stress response, functions of 97 genes were introduced (Yamamoto et al., 2012). Stomatal conductance is one of the major mechanisms for conferring the drought tolerance in vegetative organs and functions of at least eight genes are related to this mechanism. Phytochrome B (PhyB) is negatively involved in drought tolerance through the regulation of stomatal density (Liu J. et al., 2012). Calmodulin-like (CML) genes are known to regulate plant responses to abiotic stresses including drought. Transgenic plants over-expressing *OsCML4* gene confer drought tolerance through ROS-scavenging process (Yin et al., 2015). In addition, abscisic acid (ABA) is a representative hormone closely associated with abiotic stresses including drought and at least eight genes are involved in ABA relating drought stress responses. Of them, *ABA responsive AP2-like gene 1* (*ARAG1*) is related to drought tolerance during seedling stage and ABA sensitivity during germination (Zhao et al., 2010). In addition, the correlation between stomatal response and ABA is very important for studying the mechanism on the drought tolerance (Daszkowska-Golec and Szarejko, 2013). Root architecture is closely related to how vulnerable a plant will be to drought stress. Functions of a few genes have been reported in the association with both drought stress and root development. Of them, *DEEPER ROOTING 1* (*DRO1*) regulates cell elongation at the root tip and increased expression of *DRO1* changes the angle of growth so that roots develop in a more downward direction (Uga et al., 2013). Furthermore, root-specific overexpression of *OsNAC10* enlarges the roots, enhancing drought tolerance in field-grown transgenic plants and significantly increasing their grain yields under deficit conditions (Jeong et al., 2010). Thus, root-driven drought tolerance is more effective for current and future applications but our knowledge on this process is still limited.

Genome-wide transcriptome analysis is very general and powerful tool to quickly improve global understanding on this stress response. Until now, 18 series of whole genome transcriptome analyses have been performed using microarray or RNA-seq technologies. Of these, only one experiment analyzed transcriptomes in roots exposed to drought in two growth stages, tillering and panicle elongation stages, due to the difficulty of the root sampling under drought stress (Wang et al., 2011). However, physiological features of the root samples which is required for quality check of the samples used for the transcriptome analyses were not well-evaluated, limiting further applications. In addition, the detailed data analysis including *in planta* validation of the gene expression patterns, functional validation using mutants, functional classification, protein-protein interaction network, and integrating omics analysis were not provided (Chandran and Jung, 2014).

To monitor changes in gene expression when rice roots are exposed to water-deficiency stress under soil condition, we conducted RNA-Seq analysis, comparing roots samples under 2–3 d of induced stress vs. those from the well-watered (unstressed) control. The metabolic/regulatory pathways and biological processes for coping with this challenge were explored via Gene Ontology (GO) enrichment and MapMan analyses. We also examined the activity promoters of genes induced by drought conditions, using the *GUS* reporter system and developed a functional gene network to quickly understand the regulatory pathway. Functional significance for drought tolerance in root of our candidate genes is evaluated through the analysis of *osphyb* mutant carrying a T-DNA insertion in the coding sequence region, suggesting novel regulatory mechanism for the drought tolerance in rice.

## Materials and methods

### Plant materials and stress treatments

Plants of japonica rice (*Oryza sativa*) cv. Chilbo were grown in plastic pots for 4 weeks in an incubator [14-h light/10-h dark, 28°C (day)/22°C (night), humidity 80%; Younghwa Science, Daegu, Korea]. On average, 300 g of dried soil was used to grow 10 plants in each pot. The effects of drought stress were measured at various time points, including 1, 2, 3, and 4 d after irrigation was withheld and then at 7 d after they were re-watered. Our mock treatment comprised a group of rice plants that continued to receive normal irrigation throughout the experimental period. To observe the physiological features between *phyb* mutants and wild type segregants, we used samples collected before water deficiency treatment (WD), 3 d after WD, and 7 d after re-watering. In three time points, we observed the root morphology of *phyb* mutants and wild type segregants.

### RNA-seq analysis

We used the illumina platform to generate sequence reads (~26 GB) that comprised six transcriptome samples from the 2-d to 3-d drought-stressed plants plus the untreated control. In all, 100-bp paired-end reads were assessed with a FastQC toolkit (Andrews, 2010). Any adapter contaminations and low-quality reads (-phred33 and -q 20) were removed using both Cutadapt (Martin, 2011) and its wrapper tool, Trimgalore (Krueger, 2012). The resultant high-quality reads were taken for our TopHat pipeline, as described (Trapnell et al., 2013). On average, 94% of the filtered reads were mapped to the International Rice Genome Sequencing Project (IRGSP) 1.0 reference genome (Kawahara et al., 2013) and the gene features were estimated based on the gff3 annotation file provided in the Rice Genome Annotation Project (RGAP) database (http://rice.plantbiology.msu.edu/; Ouyang et al., 2007). Differentially expressed genes (DEGs) were evaluated by using Cuffdiff to compare between treatment conditions. Genes with *p* < 0.05 and log_2_ fold-changes >1 (i.e., fold-change >2) were considered differentially expressed. Further screening among the initial DEGs was done based on fragments per kilo-base per million fragments mapped (FPKM) values (Trapnell et al., 2013). The GEO accession number is GSE92989.

### GUS assays

To examine *GUS* expression patterns, we germinated seeds from two promoter trap lines in a Murashige and Skoog (MS) medium for 7 d. The resultant plantlets were then air-dried for 0.0, 0.5, 1.0, 2.0, or 4.0 h. Afterward, whole seedlings from all treatment groups were soaked for 30 min in a *GUS*-staining solution before their roots were photographed with a camera (Canon EOS 550D; Canon, Tokyo, Japan).

### Analysis of *cis*-acting elements

To identify any consensus *cis*-acting regulatory elements (CREs) in the promoters of our drought-inducible genes, we extracted 2-kb upstream sequences of ATG for *LOC_Os04g52290* and *LOC_Os07g02710* that had been validated through *GUS* assays in the current study plus those of *LOC_Os09g35790* and *LOC_Os02g0*4650, which have previously been reported as drought-inducible promoters based on the promoter-*GUS* system (Rerksiri et al., 2013; Jeong and Jung, 2015) from PLANTPAN (http://plantpan2.itps.ncku.edu.tw/; Chang et al., 2008). MEME searches were then performed with those sequences in the FASTA format via the Web server hosted by the National Biomedical Computation Resource (http://meme-suite.org/). We looked for up to four CREs with 10 maximum motif widths. Using the Motif Alignment and Search Tool (MAST) tool, we then searched DNA sequences for matches to the putative TF-binding site motifs found by the motif comparison tool (TOMTOM) within a set of promoter sequences (Bailey et al., 2006).

### Analysis of gene ontology enrichment

We employed the GO enrichment tool (Cao et al., 2012) to determine the biological roles of selected genes listed in the Rice Oligonucleotide Array Database (Jung et al., 2008b). This included any genes that were up-regulated during the 3 d of WD treatment. A fold enrichment value higher than standard (1) meant the selected GO term was over-represented. Terms with >2-fold enrichment values were also considered.

### MapMan analysis

The rice MapMan classification systems covers 36 BINs, each of which can be extended in a hierarchical manner into subBINs (Usadel et al., 2005; Urbanczyk-Wochniak et al., 2006). Using diverse MapMan tools, a significant gene list selected from high-throughput data analysis can be integrated to diverse overviews. Here, we generated a dataset carrying locus IDs from RGAP in addition to average log_2_ fold-change data for WD vs. mock treatment (control) conditions. For describing any genes up-regulated in response to 3 d of drought, we used four overviews: Metabolism, Regulation, Transcription, and Proteasome.

### Analysis of rice genes with known functions

To evaluate the functional significance of our candidate genes, we compared our gene list with the Overview of functionally characterized Genes in Rice Online database (OGRO, http://qtaro.abr.affrc.go.jp/ogro), which summarize rice genes with known functions (Yamamoto et al., 2012).

### Analysis of a predicted protein–protein interaction network

Using the Rice Interactions Viewer tool (http://bar.utoronto.ca/interactions/cgi-bin/rice_interactions_viewer.cgi; Chandran and Jung, 2014), we generated a hypothetical protein–protein interaction network involving transcription factors (TFs), kinases, transporters, and functionally characterized genes. The network was edited with the Cytoscape tool (3.2.0 version; Shannon et al., 2003).

### H_2_O_2_ measurement and antioxidant enzyme assay

Four-week-old seedlings of *phyb* mutants and wild type (WT) segregants (control) were subjected to drought stress for 3 d (drought-stressed) and then grown for 7 d after they were re-watered (recovered). Recovered, drought-stressed and control root samples were collected, and immediately frozen in liquid nitrogen and stored at −80°C. H_2_O_2_ measurement, activity of ascorbate peroxidases (APX) and catalase (CAT) enzymes were performed as described previously (Garg et al., 2012). All experimental data were recorded as the means of three independent experiments.

### Quantitative real-time PCR (qRT-PCR) analysis

Our quantitative real-time PCR (qRT-PCR) analysis was conducted as follows. Roots were sampled from WT and *phyb* mutant plants at 4 weeks old and immediately frozen in liquid nitrogen. After total RNAs were isolated using RNAiso kits (Takara Bio, Shiga, Japan), first-strand cDNA was synthesized with MMLV Reverse Transcriptase (Promega, WI, USA) and the oligo(dT) 15 primer. Synthesized cDNAs were amplified using a SYBR Premix Ex Taq (TaKaRa) before qRT-PCR was performed on a Rotor-Gene Q instrument system (Qiagen, Hiden, Germany). For normalizing the amplified transcripts, we used a primer pair for rice *ubiquitin 5 (OsUbi5/Os01g22490;* Jain et al., 2006). All primers for these analyses are summarized in Table S1.

## Results

### Physiological responses of rice roots exposed to a water deficiency

**Figure 1:**
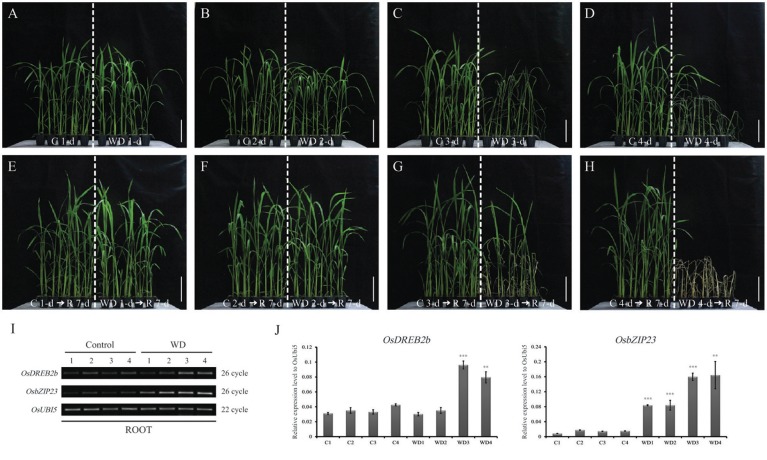
**Physiological responses under drought stress of rice seedlings grown in soil**. Rice cultivar Chilbo grown in plastic pots for 4 weeks was exposed to water deficiency (WD) for 1 **(A)**, 2 **(B)**, 3 **(C)**, or 4 **(D)** d and then recovered for 7-d **(E–H)**. Effects of WD were checked by monitoring expression patterns of drought-stress marker genes **(I,J)**. Y-axis indicates expression level relative to OsUBI5/Os01g22490 (internal control); x-axis, samples used for qRT-PCR **(J)**. C1, C2, C3, and C4, untreated control (roots from well-watered pots) corresponding to plants under 1 d of water deficiency (WD1d), 2 d (WD2d), 3 d (WD3d), and 4 d (WD4d). ^**^P < 0.01; ^***^P < 0.001. Scale bar = 10 cm. *N* = 3.

**Figure 2:**
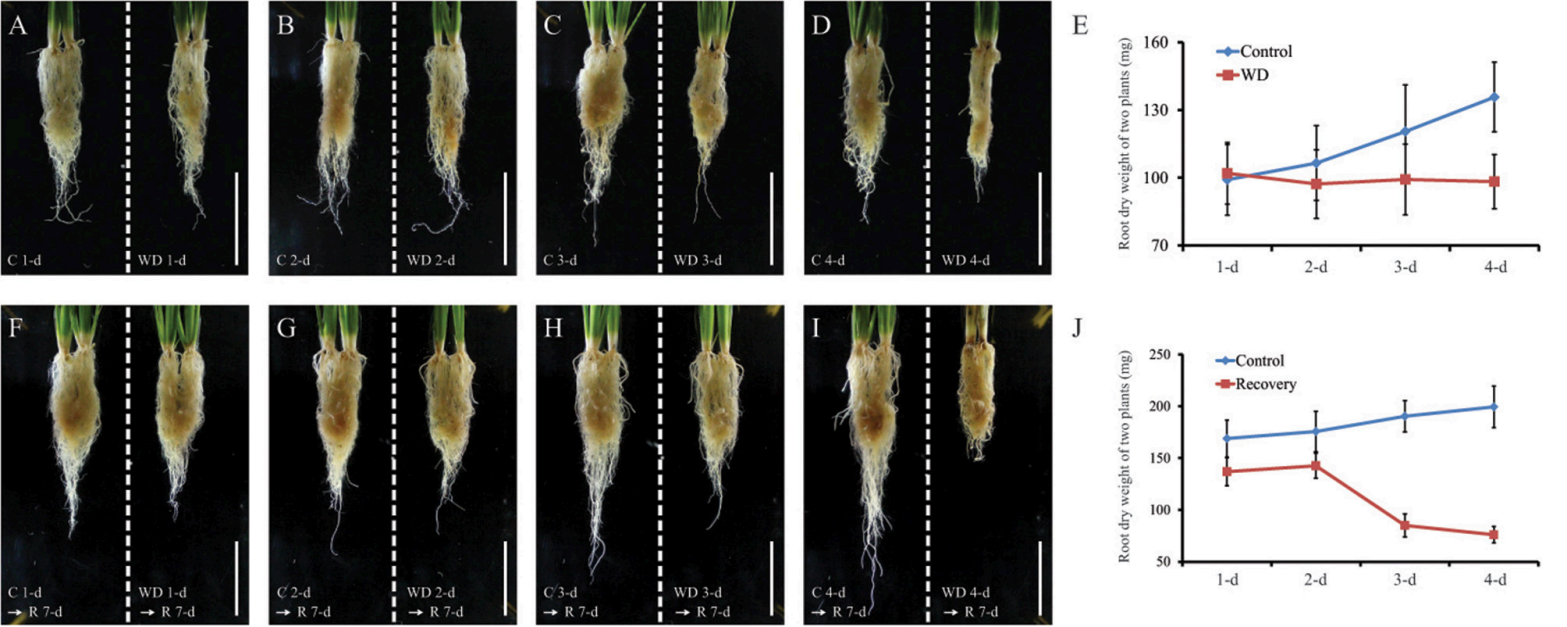
**Physiological responses under drought stress of rice roots grown in soil**. The plants grown for 4 weeks was exposed WD for 1, 2, 3, or 4 d and the dry weight was measured in roots **(A–E)**. After 7-d of recovery, dry weight was measured **(F–J)**. C1, C2, C3, and C4, untreated control (roots from well-watered pots) corresponding to plants under 1 d of water deficiency (WD1d), 2 d (WD2d), 3 d (WD3d), and 4 d (WD4d). Scale bar = 5 cm. *N* = 3 for **(A–D, F–I)**; and *n* = 200 for **(E,J)**.

Four-week-old rice seedlings grown in an incubator were exposed to a water deficit for 1, 2, 3, or 4 d (Figures 1A–D). After then, we rewatered rice seedlings in all stressed conditions and had grown them for 7 d. Most of the rice plants that were drought-treated for 2 days (2 WD) were not recovered but those treated for 3 days were mostly recovered (Figures 1E–H). To identify the DEGs in root samples, we compared between the WD treatment (1–4 d) and mock treatment (control) plants. The expression patterns were then examined for two genes that had been identified as molecular markers of the drought-stress response, i.e., *OsDREB2b* (*LOC_Os05g27930*) and *OsbZIP23* (*LOC_Os02g52780*) (Xiang et al., 2008; Matsukura et al., 2010). As expected, stressed roots (from Days 3 to 4) showed increased expression of those genes (Figures 1I,J). After then, we observed the morphological features and measured the dry weight (Figures 2A–E). As a result, the dry weights of the root samples under WD were not changed, while that of untreated control is increasing. In addition, we identified that dry weight after recovery of root samples exposed to WD stress for 2 d increased, whereas that of root samples exposed to WD stress for 3 d decreased and finally the rice plants were mostly died due to the damage augmented by the stress (Figures 2F–J). This result indicates that WD stress for 3 d is a critical time point in our experimental condition to determine the survival under WD stress. Thus, it will be very important to know the mechanism how plant roots overcome unfavorable growth condition caused by long duration expose to WD stress.

### Genome-wide identification via RNA-seq of rice genes induced by a water deficiency

**Figure 3:**
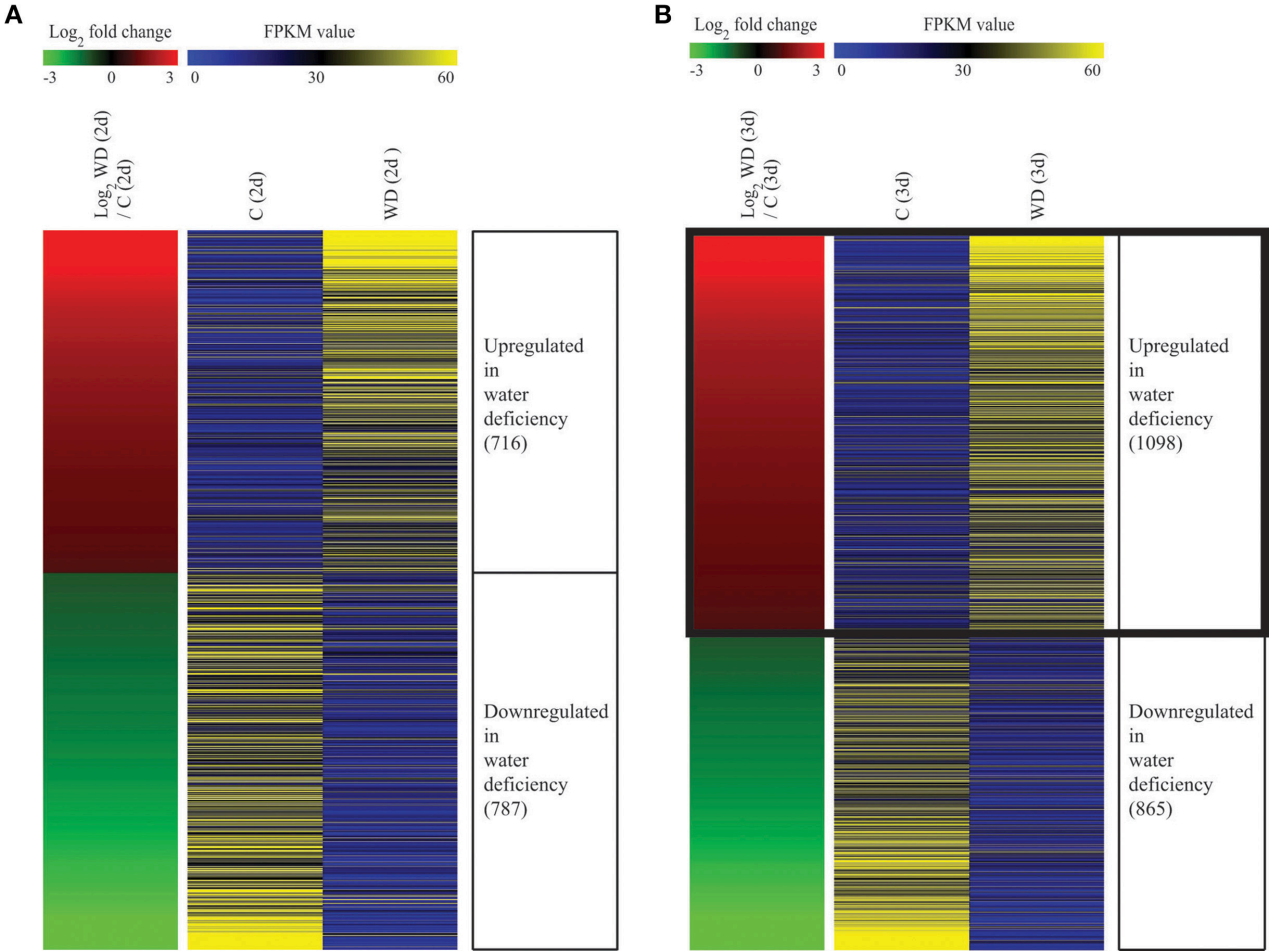
**Heatmap of differentially expressed genes under drought stress**. Using RNA-Seq data processing under criteria of FPKM > 4, *p* < 0.05, and ratio of <–1.0 for 1 <log_2_ roots exposed to water deficiency (WD) vs. mock-treated rice roots (Control, C), we identified 1,503 differentially expressed genes (DEGs) after 2 d of drought treatment **(A)** and 1,963 DEGs after 3 d of treatment **(B)**. In left panel, red color indicates upregulation in WD/C comparison; green, downregulation in WD/C comparison. Right panel shows average normalized FPKM values from RNA-Seq experiments; blue indicates lowest expression level and yellow, highest level. Detailed data about RNA-Seq analysis are presented in Tables S3, S4.

Transcriptome analysis was conducted with RNA-Seq technology using roots from control (C) and WD plants (2 or 3 d of water stress). In the 2-d treatment, 716 genes were up-regulated and 787 genes were down-regulated when compared with the control (Figure 3A, Table S2). In the 3-d trial, 1,098 genes were up-regulated and 865 genes were down-regulated (Figure 3B, Table S3). In all, 585 up-regulated genes were common to both test periods (Figure S1). For closer examination, we focused on genes that were up-regulated after 3 d of drought treatment because we had determined that this length of time is riskier for rice plants, based on their survival rates. A heat-map was constructed with data for log_2_ fold-change values between WD and control roots, and log_2_ intensities in all replicates of 1,963 DEGs, including upregulation and downregulation (Figure 3B, Table S3).

### Validation of drought-inducible genes in rice roots using GUS reporter system and qRT-PCR

**Figure 4:**
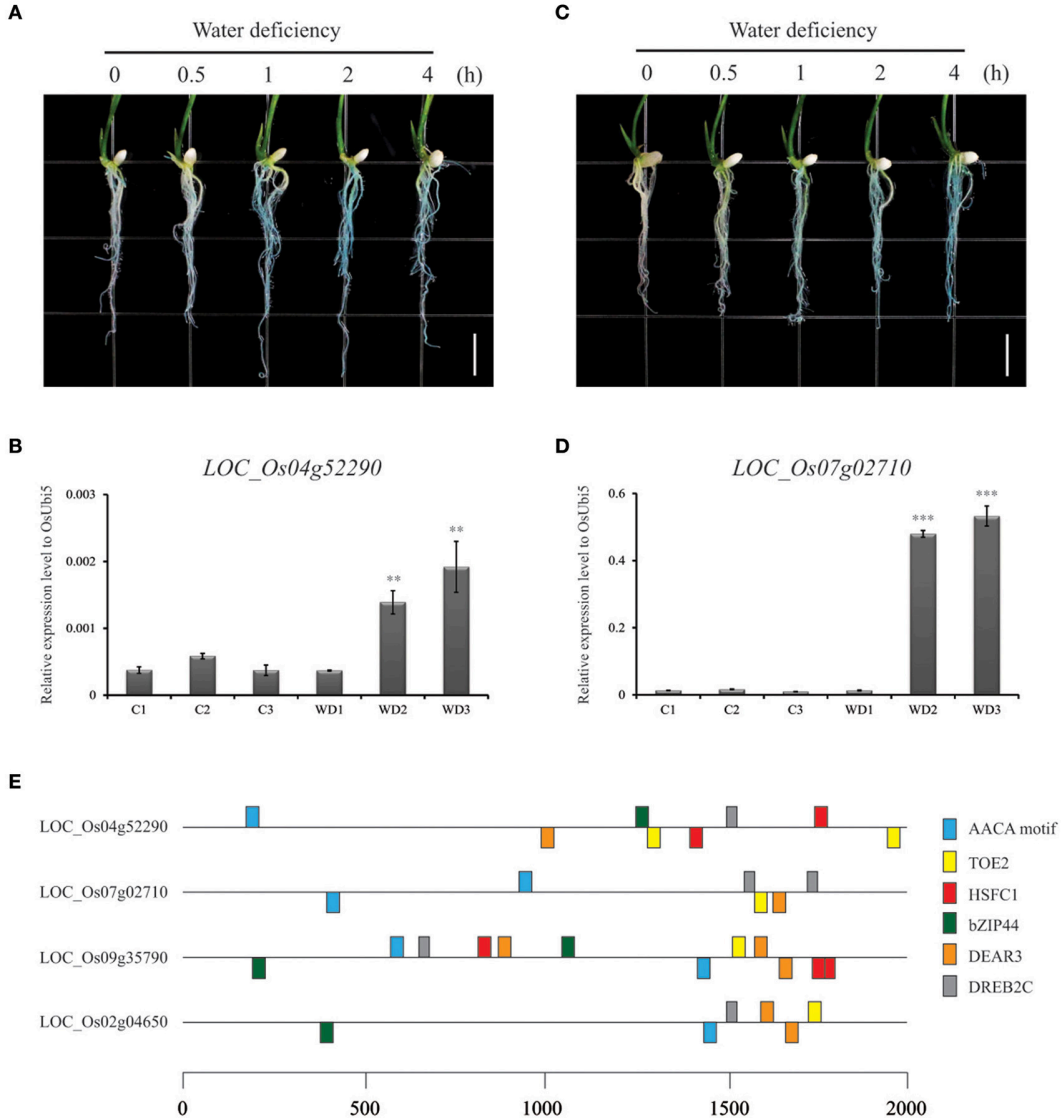
**Confirmation of water deficiency-inducible gene expression patterns in rice roots using *GUS* reporter system and qRT-PCR**. To identify *in planta* expression of selected candidates, we germinated seeds for 2 promoter trap lines (LOC_Os04g52290/3A-03417 and LOC_Os07g02710/3A-13738) in Murashige and Skoog (MS) medium for 7 d and air-dried plantlets for 0.0, 0.5, 1.0, 2.0, or 4.0 h. Whole seedlings were then incubated for 1 h in *GUS*-staining solution, respectively **(A,C)**. Expression of two genes was significantly up-regulated by WD, based on qRT-PCR **(B,D)**. C1, C2, and C3, untreated control (roots from well-watered pots) corresponding to plants under 1 d of water deficiency (WD1), 2 d of stress (WD2), and 3 d of stress (WD3). ^**^P < 0.01; ^***^P < 0.001. Identification of 6 conserved CREs of water deficiency-inducible genes **(E)**. MEME tool was used to find consensus elements in promoter regions of four genes showing *GUS* induction under water deficiency. Numerals at bottom indicate positions of nucleotides starting from ATG. Detailed information is presented in Table 1.

Promoter traps employing the *GUS* reporter gene system have been used to identify promoters involved in regulating tissue-specific and stress-responsive expression patterns (Jung et al., 2005, 2006, 2015). Our RNA-Seq data analysis revealed 98 genes showing >3 (log_2_ scale)-fold upregulation by stress when compared with the control (Table S3). We then searched the potential promoter trap lines of 38 genes and examined *GUS* expression patterns in 7-d-old seedlings. Among these, the promoter trap lines of two genes (PFG 3A-03417 for *LOC_Os04g52290* and PFG 3A-13738 for *LOC_Os07g02710*) displayed *GUS* expression in the roots after plants were exposed to a water deficit for 0–4 h (Figures 4A,C, Figure S2). We noted with interest that longer exposure to stress (i.e., 2–4 h) was associated with stronger *GUS* expression. This drought-related expression was verified by qRT-PCR (Figures 4B,D). However, the other lines did not show GUS activity, indicating that they were not real promoter trap lines. The efficiency (7.2%) showing GUS activity in the promoter trap candidate lines is a little higher than previous reports (Jeon et al., 2000; Jeong et al., 2002). Our findings demonstrated that the promoter trap system, when combined with qualified genome-wide transcriptome data, is a very effective way to identify the activity of an endogenous promoter. This also enables researchers to develop novel promoters.

### Analysis of *cis*-acting elements in two drought-inducible genes confirmed by the GUS reporter system

To identify the CREs involved in the response to a water deficit, we used two promoters from this study plus two that have shown both WD-inducible expression patterns using *GUS* reporter systems (from previous studies) as well as significant upregulation in our RNA-Seq data (Rerksiri et al., 2013; Jeong and Jung, 2015). In total, the promoters of four genes displaying significant upregulation in stressed roots were analyzed using the MEME tool (Bailey et al., 2006). From these, we found an AACA motif, a target of early activation tagged (EAT) 2 (TOE2), dehydration response element binding factor 3 (DREB3), and dehydration-responsive element-binding 2C factor (DREB2C) elements (Figure 4E, Table 1). Because they were common to all of our selected promoters, we propose that they will be crucial in future efforts to manipulate responses against water deficiencies in rice roots.

**Table 1:** **Summary of *cis*-acting regulatory elements identified in rice in response to water deficiency**.

**CREs^a^**	**Sequence**	**Function**	**References**
AACA motif	AACAAAA	Found in promoter region of cereal storage proteins	Boronat et al., 1986
TOE2	CTCCTCCTCC	TOE2 (AP2 domain)	Franco-Zorrilla et al., 2014
HSFC1	AGCTTCCAG	HSFC1 (HSF-type DNA-binding, Interferon-induced 35 kDa protein (IFP 35) N-terminus)	Franco-Zorrilla et al., 2014
bZIP44	GGCCACGTC	bZIP transcription factor binding domain	Mathelier et al., 2014
DEAR3	CCCCGCCCGC	DEAR3 (AP2 domain)	Franco-Zorrilla et al., 2014
DREB2C	CGGCCGGGCC	DREB2C (AP2 domain)	Franco-Zorrilla et al., 2014

a *Indicates cis-acting regulatory elements*.

### Analysis of gene ontology enrichment reveals biological processes associated with water-deficiency responses in rice roots

To determine the functions of 1,098 genes up-regulated by drought in rice roots, we studied the GO terms of those genes within the “biological process” category. In all, 19 terms were highly over-represented in our gene list, with *p* < 0.05 and fold-enrichment values of >2 (log_2_)-fold, as we have also previously reported (Jung et al., 2008b; Table S4).

Of these, “valyl-tRNA aminoacylation” (14.0-fold enrichment) was the most significantly enriched by drought stress. Metabolic profiling of *Pisum sativum* L. (Charlton et al., 2008) has shown that valine along with threonine, leucine, and isoleucine are important metabolites that are most highly accumulated in response to a water deficit. Moreover, the levels of branched chain amino acids, such as valine, leucine, and isoleucine are greatly increased under water stress in leaf tissues from cultivars of wheat (*Triticum aestivum*; Bowne et al., 2012). All of these results suggest that aminoacylation of valyl-tRNA might be activated during drought periods.

**Figure 5:**
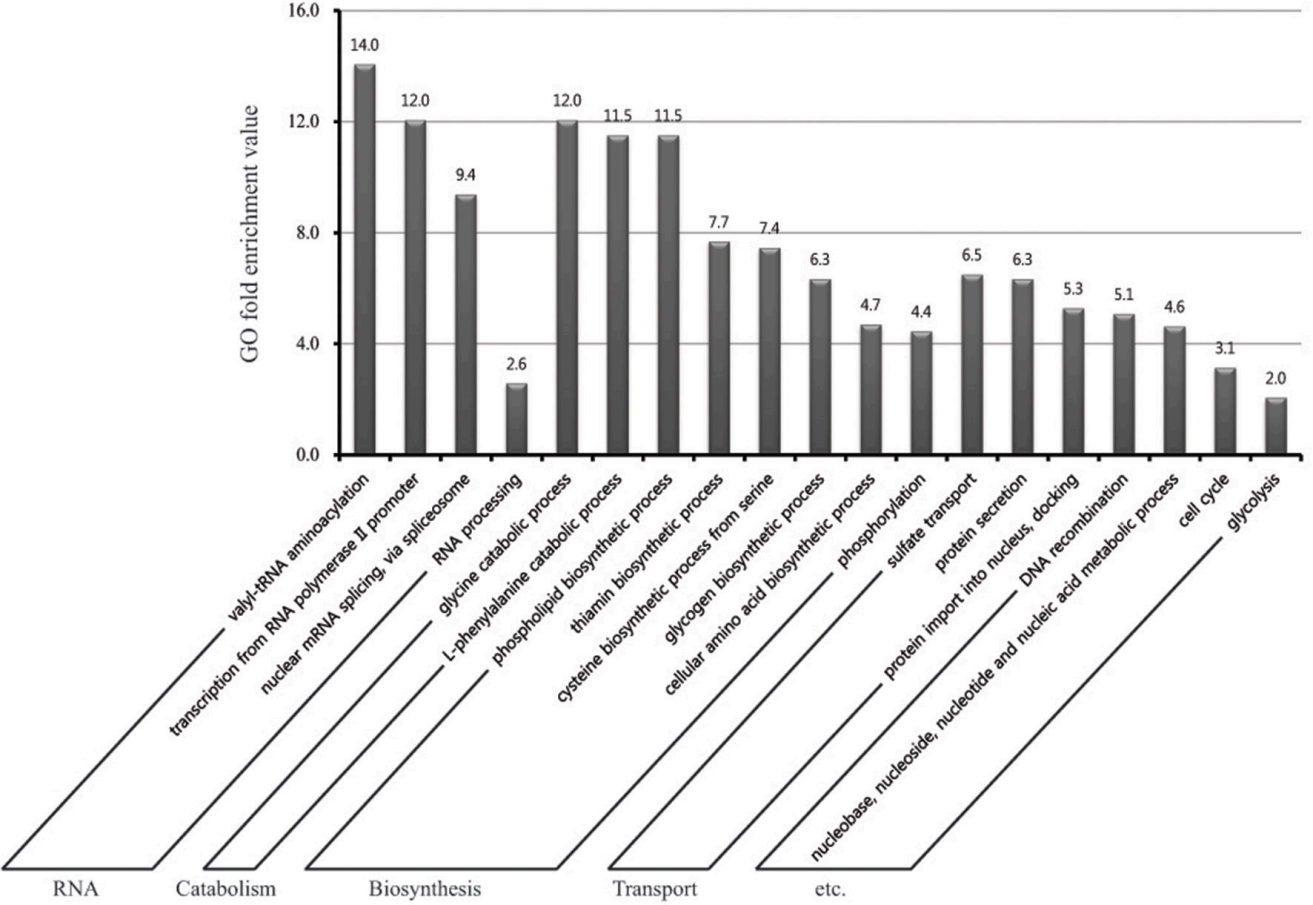
**Gene ontology (GO) enrichment analysis in “biological process” category for genes up-regulated in response to water deficiency**. In all, 19 GO terms were over-represented under >2-fold enrichment value, with *p* < 0.05. Details of GO assignments are presented in Table S5.

Genes for “phospholipid biosynthetic process” (11.5), “thiamin biosynthetic process” (7.7), “cysteine biosynthetic process from serine” (7.4), “glycogen biosynthetic process” (6.3), and “cellular amino acid biosynthetic process” (4.7) also have important roles in the abiotic-stress response (Figure 5). For example, Phospholipase C (PLC) plays a role in catalyzing hydrolysis and *AtPLC1* gene is known to be induced by drought and salt stresses (Hirayama et al., 1995). Furthermore, thiamine (vitamin B1) is up-regulated under salinity or osmotic-stress conditions by abscisic acid (ABA) in *Arabidopsis thaliana* (Rapala-Kozik et al., 2012). Transgenic *Arabidopsis* plants over-expressing *T. aestivum* cysteine protease exhibit higher drought tolerance and greater cysteine protease activity under water-stress conditions than do WT plants (Zang et al., 2010). The effects of lipids on signaling, intracellular trafficking, and cytoskeletal organization also have important roles in responses to drought and salinity (Wang et al., 2007). We also identified other significant GO terms such as “catabolic process,” “Transport,” and “etc.” that are significant in the drought response (Figure 5). Therefore, the biological processes identified here as being closely associated with WD might prove to be novel resources for improving our understanding about the molecular mechanism and components involved in conferring plant tolerance to moisture stress.

### MapMan analysis of water deficiency-related genes in rice roots

The MapMan program is very effective for visualizing diverse overviews associated with high-throughput transcriptome data (Jung and An, 2012). We uploaded fold-change data and Locus IDs for 1,098 upregulated genes (Table S3) to various overviews installed in that program.

**Figure 6:**
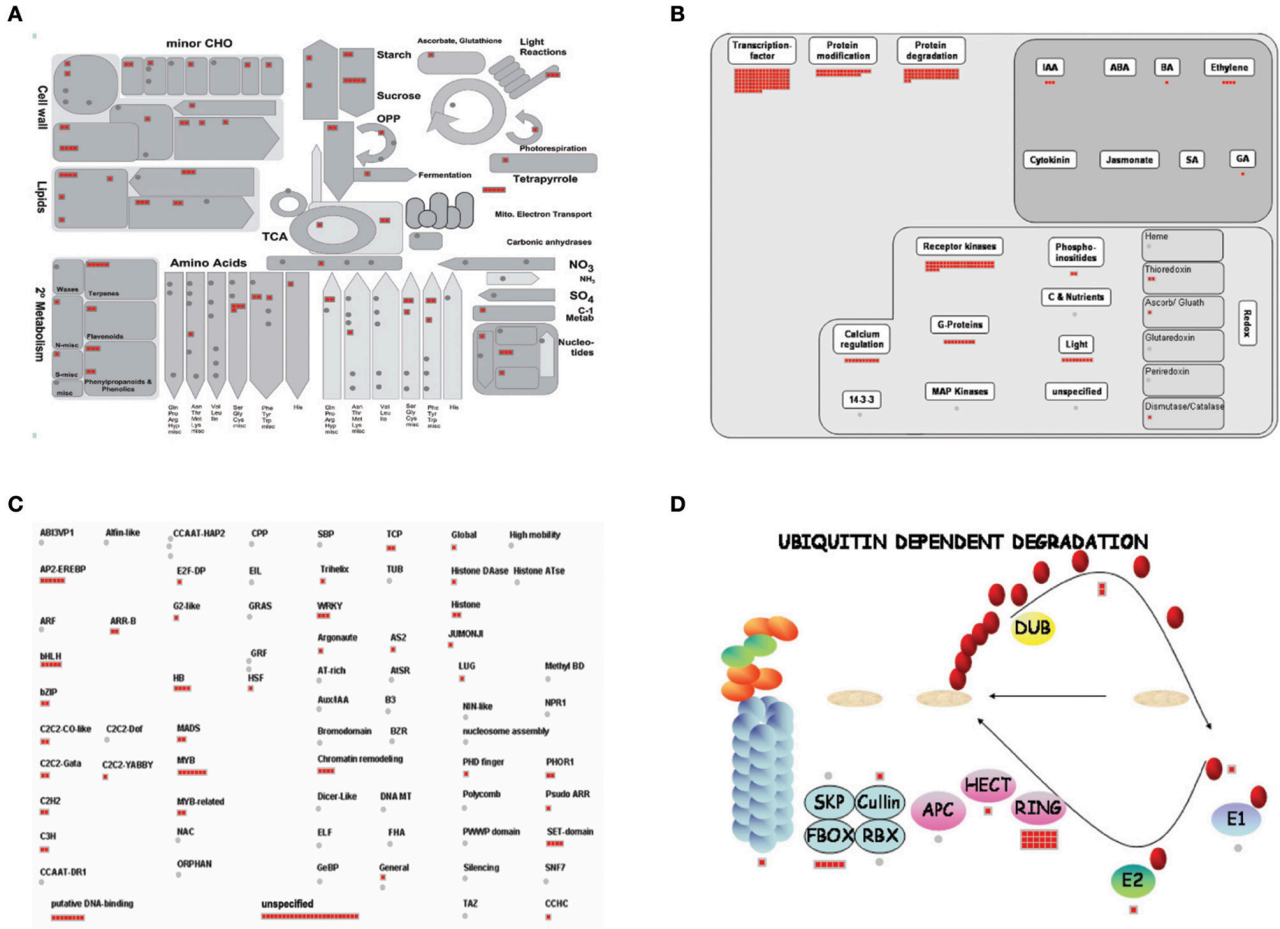
**MapMan analysis of genes associated with response to water deficiency**. Overviews: **(A)** Metabolism, **(B)** Regulation, **(C)** Transcription, and **(D)** Proteasome. Red boxes indicate genes up-regulated by stress. Detailed information is presented in Tables S6, S7.

To investigate the significant metabolic pathways involved in the response to water-deficiency stress, we analyzed the Metabolism overview associated with 1,098 genes (Figure 6). Amino acid metabolism (19 elements), lipids (16), secondary metabolism (14), cell walls (14), nucleotides (5), mitochondrial electron transport (5), photosystems (4), and the Calvin Cycle (3) were clearly related to this stress (Figure 6A, Tables S5, S6). These results implied that rice roots might trigger the above metabolic pathways to increase drought tolerance. Our Regulation overview of 1,098 genes demonstrated that 104 TFs, 29 genes related to protein modification, and 50 genes associated with protein degradation were expressed in rice roots during periods of water stress (Figure 6B). Of these, TFs were the most abundant, meaning that they are largely involved in regulating the response and tolerance of rice to drought conditions. Therefore, those genes are considered potential candidates for further study.

Accordingly, we found nine myeloblastosis (MYB) oncogenes, six Apetala2/Ethylene Responsive Element Binding Proteins (AP2/EREBPs), five Basic Helix-Loop-Helix (bHLH) genes, four homeobox genes, four Chromatin Remodeling Factors, four SET-domains, three WRKY domains, two Cys2His2 (C2H2) zinc fingers, two C3H zinc fingers, two MADS box genes, two members of the TEOSINTE BRANCHED1, CYCLOIDEA, AND PCF FAMILIES, and two basic leucine zipper (bZIP) TFs for water-deficiency stress (Figure 6C, Tables S5, S6). Dehydration-responsive element-binding protein (DREB) is part of a subfamily of the AP2/EREBP and plays a significant role in the abiotic-stress response by specifically binding to the dehydration-responsive element/C-repeat (DRE/CRT) *cis*-acting element (Mizoi et al., 2012). For example, *OsDREB1C, OsDREB1F*, and *OsDREB2A* are constitutively expressed under drought conditions (Dubouzet et al., 2003; Wang et al., 2008), and overexpression of *OsDREB1F, OsDREB1G*, and *OsDREB2A* improves tolerance to moisture stress by transgenic rice plants (Chen et al., 2008; Wang et al., 2008; Cui et al., 2011). Likewise, bZIP, MYB, and WRKY genes have been explored for their ability to improve drought tolerance in rice. *OsbZIP16, OsbZIP23, OsbZIP46, OsbZIP71*, and *OsbZIP72* have critical functions in ABA signal transduction and they positively regulate drought tolerance (Xiang et al., 2008; Lu et al., 2009; Chen et al., 2012; Tang et al., 2012; Liu et al., 2014). Rice gene *OsMYB4* has a positive role in conferring drought tolerance when expressed in transgenic plants of *Malus domestica* and *Solanum lycopersicum* (Vannini et al., 2007; Pasquali et al., 2008). As an R2R3-type MYB gene, *OsMYB2* over-expressed in plants makes them more tolerant to salt, cold, and dehydration while overexpression of *OsMYB48-1*, another MYB-related TF, increases drought and salinity tolerance in rice (Yang et al., 2012; Xiong et al., 2014). Increased expression of *OsWRKY11* under the control of *HSP101* promoter also leads to enhanced drought tolerance (Wu et al., 2009), as does overexpression of *OsWRKY45* (Tao et al., 2011). In addition, various TFs, e.g., NAC, bHLH, Zinc fingers, and homeobox genes, are involved in the plant response to water deficits (Zhang et al., 2016).

We found that the responses of RING-finger E3 ligases (18 elements), Ubiquitin E3 F-Box (5), and ubiquitin protease (2) were clearly related to drought stress (Figure 6D, Tables S5, S6). For example, OsDIS1 is a C3HC4 RING finger E3 ligase, and the RNAi transgenic plants are tolerant to drought stress (Ning et al., 2011). The RING-finger containing E3 ligase *O. sativa SALT-AND DROUGHT-INDUCED RING FINGER 1* (*OsSDIR1*) is a candidate gene for engineering drought tolerance in various crop plants. Its expression is also up-regulated by excess NaCl (Gao et al., 2011). The expression patterns of 47 OsRFP genes in response to abiotic stresses have been monitored via semi-quantitative reverse transcription PCR and *in silico* analysis (Lim et al., 2013). Our results also suggest a strong correlation between drought and activity of RING finger E3 ligase.

### Evaluation of candidate genes associated with drought stress using rice genes with known functions

To evaluate the significance of our candidate genes, we searched the literature to determine if functions for them have been reported previously. This was accomplished with the online OGRO database, which provides a thorough summary of rice genes that have been characterized through molecular and genetic techniques (Yamamoto et al., 2012). Of the 41 genes found in that database (Table 2), 18 have been linked to various abiotic-stress responses in rice. They include *PhyB* (Liu J. et al., 2012), *OsbZIP52*/*RISBZ5* (Liu C. et al., 2012), *OsETOL1* (Du et al., 2014), *trehalose-6-phosphate synthase 1* (*OsTPS1*; (Li H. W. et al., 2011), *OsTZF1* (Jan et al., 2013), and *sHSP17.7* (Sato and Yokoya, 2008) for drought; *OsCIPK15* (Xiang et al., 2007), *OsGAPC3* (Zhang et al., 2011), *glyoxalase I* (*OsGLYI-11.2*; Mustafiz et al., 2014), *Programmed cell death 5* (*OsPDCD5*; Yang et al., 2013), OsPLDα1 (Shen et al., 2011) *OsTPS1* (Li H. W. et al., 2011), and *OsTZF1* (Jan et al., 2013), for salinity; *OsbZIP52*/*RISBZ5* (Liu C. et al., 2012) *OsLti6b* (Kim et al., 2007), *OsTPS1* (Li H. W. et al., 2011), and *v1* (Kusumi et al., 2011) for chilling; and *ETHYLENE OVERPRODUCER 1-like* (*OsETOL1*) for tolerance to submergence (Du et al., 2014; Table 2). These findings indicate that our candidate genes are potentially involved in plant responses to abiotic stress (including drought) and suggest the possibility of crosstalk among related pathways.

**Table 2:** **Functionally characterized drought-related genes from rice previously reported in literature**.

**Major category^a^**	**Minor category^b^**	**Locus_id**	**Gene name**	**Gene symbol**	**Method^c^**	**DOI references^d^**
RT^e^	Drought	LOC_Os03g19590.1	Phytochrome B	phyB	M	10.1007/s11103-011-9860-3
RT	Drought	LOC_Os06g45140.1	Basic leucine zipper 52	OsbZIP52/RISBZ5	OX	10.1007/s00425-011-1564-z
RT	Drought	LOC_Os03g18360.1	ETHYLENE OVERPRODUCER 1-like	OsETOL1	KD OX	10.1111/tpj.12508
RT	Drought	LOC_Os05g44210.1	Trehalose-6-phosphate synthase1	OsTPS1	OX	10.1007/s00425-011-1458-0
RT	Drought	LOC_Os05g10670.1	Tandem zinc finger 1	OsTZF1	KD OX	10.1104/pp.112.205385
RT	Drought	LOC_Os03g16040.1	Small heat-shock protein17.7	sHSP17.7	OX	10.1007/s00299-007-0470-0
RT	Salinity	LOC_Os11g02240.1	Calcineurin B-like protein-interacting protein kinase15	OsCIPK15	OX	10.1104/pp.107.101295
RT	Salinity	LOC_Os08g03290.1	Cytosolic GAPDH protein 3	OsGAPC3	OX	10.1007/s11240-011-9950-6
RT	Salinity	LOC_Os08g09250.1	Glyoxalase1	OsGLY1-11.2	Others	10.1111/tpj.12521
RT	Salinity	LOC_Os05g47446.1	Programmed cell death 5	OsPDCD5	KD	10.1007/s11032-012-9793-9
RT	Salinity	LOC_Os01g07760.1	Phospholipase Dα1	OsPLDα1	KD	10.1111/j.1744-7909.2010.01021.x
RT	Salinity	LOC_Os05g44210.1	Trehalose-6-phosphate synthase1	OsTPS1	OX	10.1007/s00425-011-1458-0
RT	Salinity	LOC_Os05g10670.1	Tandem zinc finger 1	OsTZF1	KD OX	10.1104/pp.112.205385
RT	Cold	LOC_Os06g45140.1	Basic leucine zipper 52	OsbZIP52/RISBZ5	OX	10.1007/s00425-011-1564-z
RT	Cold	LOC_Os05g04700.1	OsLti6b	OsLti6b	OX	10.1007/s00299-006-0297-0
RT	Cold	LOC_Os05g44210.1	Trehalose-6-phosphate synthase1	OsTPS1	OX	10.1007/s00425-011-1458-0
RT	Cold	LOC_Os03g45400.1	Virescent-1	v(1)	M	10.1111/j.1365-313X.2011.04755.x
RT	Submergency	LOC_Os03g18360.1	ETHYLENE OVERPRODUCER 1-like	OsETOL1	KD OX	10.1111/tpj.12508
RT	Blast	LOC_Os05g31140.1	β-glucanase1	Gns1	OX	10.1023/A:1020714426540
RT	Blast	LOC_Os12g03040.1	ONAC131	ONAC131	KD	10.1007/s11033-012-2040-y
RT	Blast	LOC_Os12g03040.1	ONAC131	ONAC131	KD	10.1007/s11103-012-9981-3
RT	Blast	LOC_Os03g19590.1	Phytochromeb	phyB	M	10.1093/mp/ssr005
RT	Blast	LOC_Os01g49290.1	Receptor for Activated C-Kinase 1A	RACK1A	OX	10.1105/tpc.107.054395
RT	Soil stress	LOC_Os05g03780.1	Metal Tolerance Protein 1	OsMTP1	KD OX	10.1007/s00299-011-1140-9
RT	Soil stress	LOC_Os07g15460.1	Natural resistance-associated macrophage protein1	OsNRAMP1	OX	10.1093/jxb/err136
RT	Soil stress	LOC_Os08g10630.1	Zn-regulated transporter, iron (Fe)-regulated transporter-like protein4	OsZIP4	OX	10.1093/jxb/erm147
RT	Other stress	LOC_Os08g09250.1	Glyoxalase1	OsGLY1-11.2	Others	10.1111/tpj.12521
RT	Other stress	LOC_Os09g20284.1	Polyamine oxidases 7	OsPAO7	Others	10.1093/pcp/pcu047
RT	Other stress	LOC_Os03g16040.1	Small heat-shock protein17.7	sHSP17.7	OX	10.1023/B:MOLB.0000018764.30795.c1
MT^f^	Dwarf	LOC_Os06g02019.1	Ent-kaurenoic acid oxidase	oskao	M	10.1104/pp.103.033696
MT	Dwarf	LOC_Os06g06050.1	Dwarf 3	d3	M	10.1093/pcp/pci022
MT	Dwarf	LOC_Os06g12400.1	Rice homeobox gene 1a	HOX1a	OX	10.1111/j.1744-7909.2011.01075.x
MT	Dwarf	LOC_Os04g01590.1	OsARG	OsARG	M	10.1111/j.1365-313x.2012.05122.x
MT	Dwarf	LOC_Os01g03510.1	Actin-interacting protein 1	AIP1	KD OX	10.1111/tpj.12065
MT	Root	LOC_Os07g48560.1	WUSCHEL-related Homeobox 11	wox11	M	10.1105/tpc.108.061655
MT	Root	LOC_Os06g06050.1	Dwarf3	d3	M	10.1007/s00344-011-9228-6
MT	Root	LOC_Os01g03510.1	Actin-interacting protein 1	AIP1	KD OX	10.1111/tpj.12065
MT	Culm leaf	LOC_Os06g06050.1	Dwarf 3	d3	M	10.1093/pcp/pci022
MT	Culm leaf	LOC_Os03g19590.1	Phytochrome B	phyB	M	10.1007/s11103-011-9860-3
MT	Seed	LOC_Os02g52480.1	Kip-related protein1	KRP1	OX	10.1104/pp.106.087056
MT	Seed	LOC_Os04g01590.1	OsARG	OsARG	M	10.1111/j.1365-313x.2012.05122.x
MT	Shoot seedling	LOC_Os03g19590.1	Phytochrome B	phyB	M	10.1093/pcp/pcs097
MT	Shoot seedling	LOC_Os06g06050.1	Suppressors of lazy1	SOL1	M	10.1073/pnas.1411859111
MT	Panicle flower	LOC_Os04g01590.1	OsARG	OsARG	M	10.1111/j.1365-313x.2012.05122.x
PT^g^	Source activity	LOC_Os01g12710.1	Non-yellow coloring1	nyc1	M	10.1105/tpc.106.042911
PT	Source activity	LOC_Os01g09620.1	Delay of the onset of senescence	OsDOS	KD OX	10.1104/pp.106.082941
PT	Source activity	LOC_Os05g10670.1	Tandem zinc finger 1	OsTZF1	KD OX	10.1104/pp.112.205385
PT	Source activity	LOC_Os04g57850.1	SLOW ANION CHANNEL-ASSOCIATED 1	SLAC1	M	10.1093/jxb/ers216
PT	Sterility	LOC_Os04g01590.1	OsARG	OsARG	M	10.1111/j.1365-313x.2012.05122.x
PT	Sterility	LOC_Os02g55570.1	Shugoshin protein1	Ossgo1	M	10.1111/j.1365-313X.2011.04615.x
PT	Sterility	LOC_Os12g42760.1	OsSpo11-4	OsSpo11-4	KD	10.1371/journal.pone.0020327
PT	Sterility	LOC_Os05g05280.1	POLLEN TUBE BLOCKED 1	PTB1	M	10.1038/ncomms3793
PT	Sterility	LOC_Os09g27620.1	PERSISTANT TAPETAL CELL1	PTC1	M	10.1104/pp.111.175760
PT	Sterility	LOC_Os06g02019.1	Reduced pollen elongation1	rpe1	M	10.1105/tpc.107.054759
PT	Sterility	LOC_Os09g27620.1	Thermo-sensitive genic male sterility 9-1	TMS 9-1(OsMS1)	NV	10.1007/s00122-014-2289-8
PT	Eating quality	LOC_Os09g29404.1	Isoamylase3	isa3	M	10.1093/pcp/pcr058
PT	Eating quality	LOC_Os03g09250.1	Rice myo-inositol 3-phosphate synthase 1	RINO1	KD	10.1093/pcp/pcp071
PT	Eating quality	LOC_Os03g09250.1	Rice myo-inositol 3-phosphate synthase 1	RINO1	KD	10.1111/j.1467-7652.2008.00375.x
PT	Flowering	LOC_Os01g69850.1	OsMADS51	OsMADS51	M	10.1104/pp.107.103291
PT	Flowering	LOC_Os03g19590.1	Phytochrome B	phyB	M	10.1105/tpc.105.035899
PT	Flowering	LOC_Os07g49460.1	*Oryza sativa* Pseudo-response regulator37	OsPRR37	NV	10.1093/mp/sst088
PT	Panicle flower	LOC_Os03g18360.1	ETHYLENE OVERPRODUCER 1-like	OsETOL1	KD OX	10.1111/tpj.12508
PT	Culm leaf	LOC_Os01g52110.1	Rice RING zinc-finger protein 34	OsRZFP34	KD OX	10.1007/s11103-014-0217-6
PT	Seed	LOC_Os01g49290.1	Rice receptor for activated C kinase 1A	OsRACK1A	KD OX	10.1371/journal.pone.0097120
PT	Others^h^	LOC_Os03g18360.1	ETHYLENE OVERPRODUCER 1-like	OsETOL1	KD OX	10.1111/tpj.12508
PT	Others	LOC_Os08g09250.1	Glyoxalase1	OsGLY1-11.2	Others	10.1111/tpj.12521
Others^h^	Others^h^	LOC_Os05g50380.1	ADP-glucose pyrophosphorylase large subunit 3	LSU3	M	10.1071/FP12186
Others	Others	LOC_Os04g35420.1	OsRecQl4	OsRecQl4	OX	10.1093/pcp/pcs155

a *Of agronomic traits associated with functionally characterized genes out of candidate genes in this study*.

b *Indicates sub-agronomic trait categories in each of major categories*.

c *Indicates methods used for the functional characterization: M indicates mutants by T-DNA/Tos17/Ds insertion; KD, knockdown mutants by RNAi or anti-sense approaches; OX, overexpressed mutants by transgenic approaches; and NV, natural variation; and others, those by other methods besides four major methods*.

d *Indicates Digital Object Identifier (DOI)*.

e *Indicates Resistance or Tolerance relating trait*.

f *Indicates Morphological trait*.

g *Indicates Physiological trait*.

h *Indicates other agronomic traits besides determined major or minor categories*.

### Analyses of predicted protein–protein interactions associated with drought tolerance

**Figure 7:**
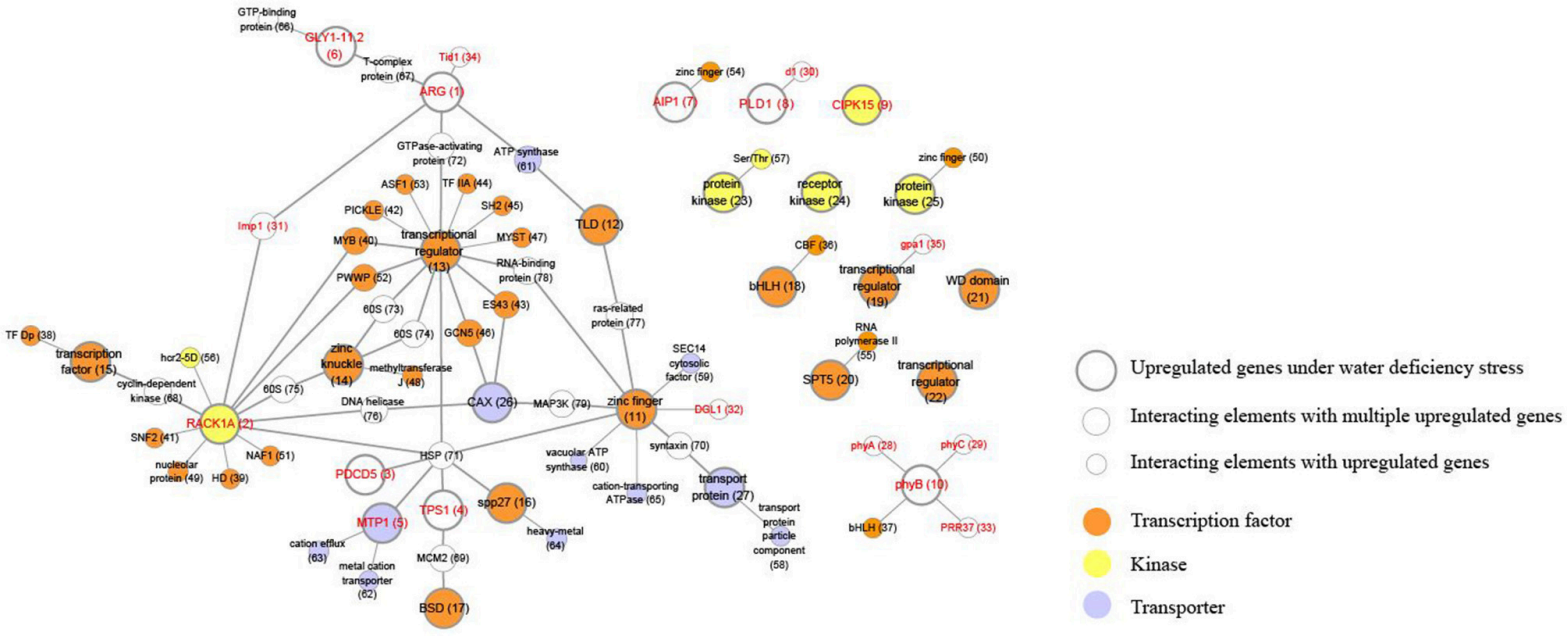
**Construction of regulatory network associated with genes up-regulated under WD**. Using Rice Interaction Viewer and Cytoscape tools, we queried predicted protein–protein interaction network associated with functionally characterized genes, kinase, transcription factors, and transporters, and identified 10 functionally characterized genes (red lettering in large circle/nodes), 12 TFs (large brown circles/nodes), 4 kinases (large yellow circles/nodes), and 3 transporters (large sky-blue circles/nodes) in network. Furthermore, 32 elements (small circles) interact with upregulated genes under WD (large circles); 20 elements interacting with multiple upregulated genes are indicated by medium-sized nodes and thick lines. Detailed information about values in round brackets of nodes is presented in Table S8.

Regulatory genes are primary targets when investigating diverse stress responses and developmental processes. Among the 1,098 genes that were up-regulated by 3 d of drought treatment, we identified 104 TFs, 44 kinases, 27 transporters, and 40 functionally characterized genes (Figure 7, Table 2, Table S6). Understanding the regulatory relationships among them can improve our ability to develop novel strategies for enhancing plant tolerance to various stresses. To increase our knowledge, we utilized the Rice Interactions Viewer to generate a hypothetical protein–protein interaction network associated with those up-regulated genes mentioned above (Ho et al., 2012). Four categories were used as query in the refined network: 10 functionally characterized genes (red lettering in large circles of Figure 7), 12 TFs (large brown circles), four kinases (large yellow circles), and three transporters (large sky-blue circles). We also highlighted elements with multiple interactions among these regulatory and functionally characterized genes. Details about locus IDs, gene names, and putative functions are provided in Table S7.

### Drought tolerance of *phyb* mutant through effective reactive oxygen species (ROS) scavenging in roots

**Figure 8:**
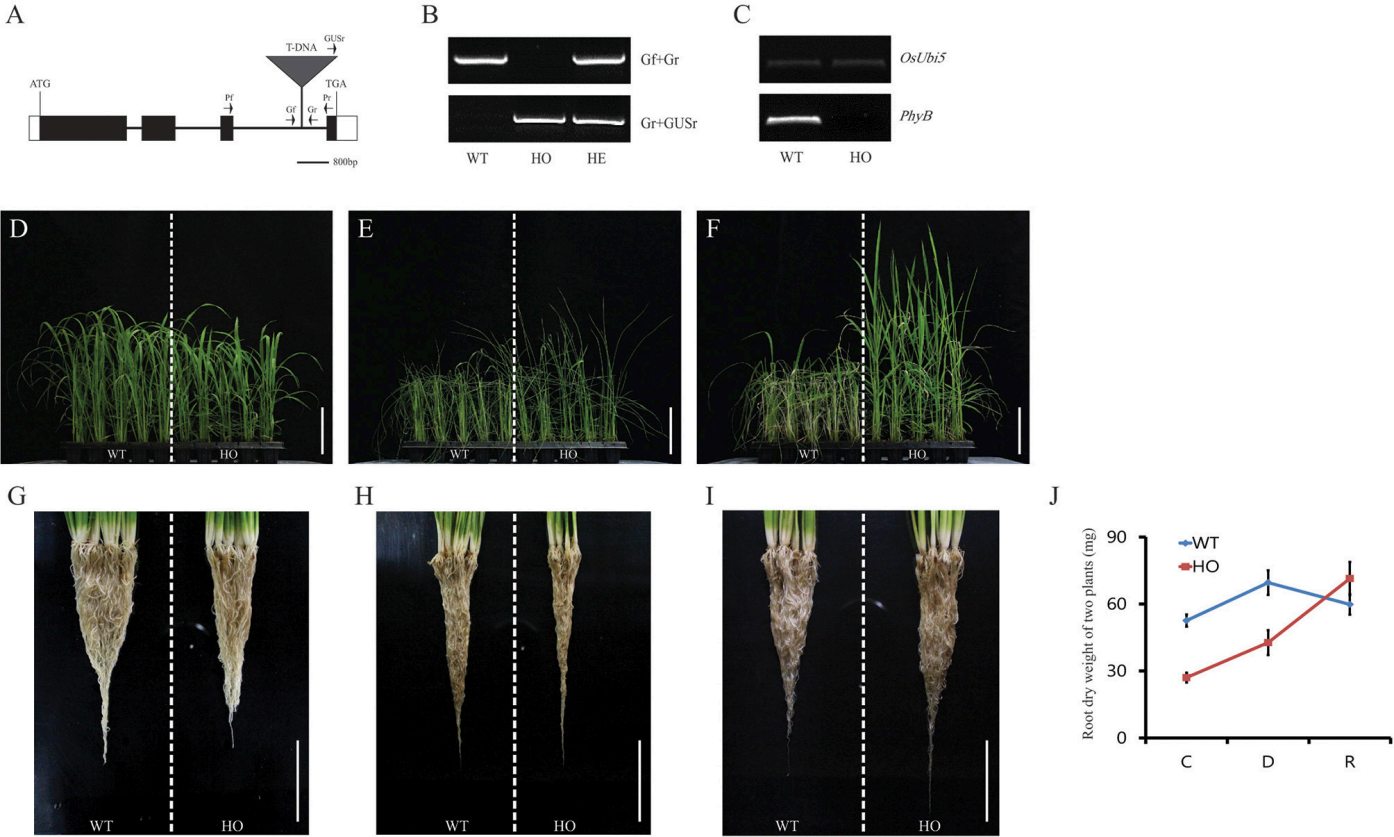
**Drought-stress response mediated by *PhyB* using T-DNA insertional mutant**. Schematic diagram for T-DNA insertion site in *PhyB* of Line 4A-02226 is shown **(A)**. Black boxes represent exons; white boxes, UTR; lines between boxes, introns; gray triangle, T-DNA insertion; small arrows, gene-specific primers for qRT-PCR analysis and genotyping of tagged gene. ATG and TGA indicate start and stop codons. Scale bar = 800 bp. Genotyping was performed for *phyB* mutant line **(B)**. Genotyping experiment identified homozygous progenies with T-DNA in *PhyB*. WT, wild-type segregants of T-DNA insertional line in *PhyB*; HO, homozygote; HE, heterozygote. Expression of *PhyB* was significantly suppressed in mutant **(C)**. *OsUBI5 (LOC_Os01g22490)* was used as internal control. *phyB* homozygous progenies with T-DNA insertion demonstrated drought-tolerant phenotype. *phyB* and wild-type segregants grown in plastic pots for 4 weeks was exposed to water deficiency for 3 d. Photos were taken 7 d after re-watering **(D–F)**. Scale bar = 10 cm. **(G–J)** shows the respective roots and dry weight. Scale bar = 5 cm. *N* = 3 for **(D–I)**; *n* = 100 for **(J)**.

Of the 41 genes with known functions, alleles with T-DNA insertions were found for mutants of *phytochrome B (phyb)*/*LOC_Os03g19590* (Figure 7, number 10). As we have reported previously (Liu J. et al., 2012), a drought-tolerant phenotype has been confirmed in 30-d-old greenhouse-grown seedlings of *phyb* mutant Line 4A-02226, which has a T-DNA insertion in the 3rd intron (Figures 8A–F). This study informed that drought tolerance in *phyb* mutant is caused by reduction of stomatal density but not by root morphology. However, in our study, the comparison of dry weight between WT and *phyb* mutant roots exposed to WD stress for 3 d revealed that *phyb* mutant roots have 48.5% less dry weight than that of WT. More interestingly, after recovery process, WT did not survive and the dry weight of the root was reduced, while *phyb* mutant survived and the dry weight of roots even increased due to the continuous growth of the roots (Figures 8G–J). This result indicates that root development is very important for the drought stress tolerance and also we are interested in the mechanism how rice roots overcome damage caused by prolonged WD stress. It was known that processing of reactive oxygen species (ROS) play important roles in root development of other plant species (Causin et al., 2012; Zhao et al., 2016).

**Figure 9:**
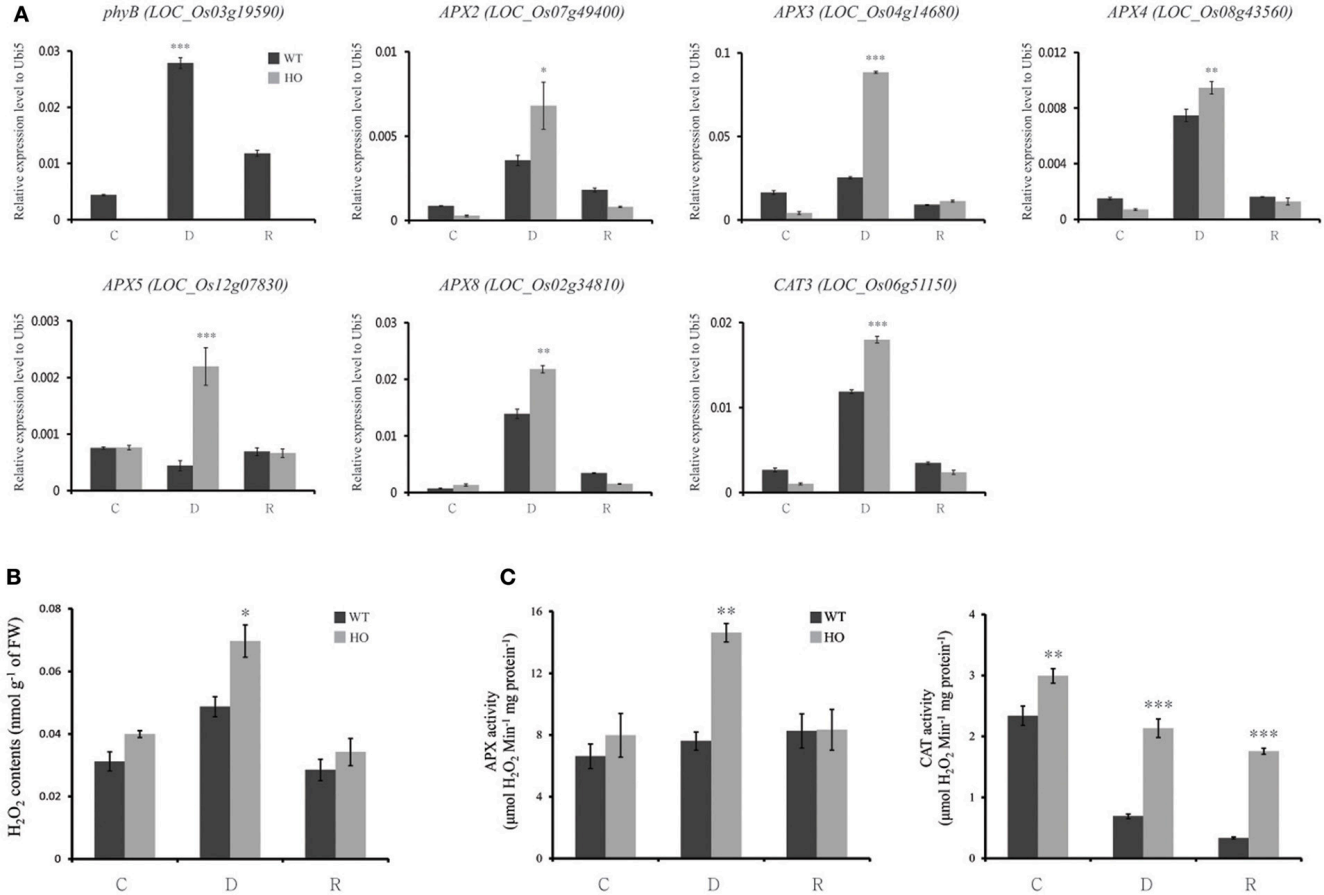
**Comparison of transcripts of ROS-relating genes, H_2_O_2_ contents, and ROS-scavenging enzymatic activity between WT and *phyB* mutant**. Analyses of transcripts of *phyB, APX2, APX3, APX4, APX5, APX8*, and *CAT3* **(A)**, H_2_O_2_ contents **(B)** and enzymatic activity of APX and CAT **(C)** in roots of WT and *phyB* (HO) mutant under before drought (control), after drought and recovery. The expression levels were normalized to that of *Ubi5* using real-time polymerase chain reaction **(A)**. As the biological replicates, we used different three samples. The error bars of the each sample means ± *SD* (*n* = 9) **(B,C)**. WT, wild-type segregants of T-DNA insertional line in *PhyB*; HO, homozygote; C, control; D, drought treatment for 3 d; R, recovery for 7 d after drought treatment. ^*^P < 0.05; ^**^P < 0.01; ^***^P < 0.001.

Several studies have reported a correlation between major active enzyme activity and drought (Huang et al., 2010; Huda et al., 2013; Xu et al., 2016). The enzyme-catalyzed reactions are the main mechanisms for the removal of superoxide and H_2_O_2_. Especially, ascorbate peroxidases (APXs) and catalases (CATs) are known to be important enzymes involved in H_2_O_2_ removal under drought stress (Noctor et al., 2014). Therefore, we analyzed the expression patterns of CAT and APX antioxidant enzyme families using real-time PCR analyses for the samples under normal condition, drought stress condition for 3 d, and recovery after drought stress. As a result, we found that expressions of five of the eight APX genes and one of the three CAT genes were upregulated in *phyB* mutant under drought stress, suggesting the major contribution of APX to remove ROSs produced by drought stress in rice roots (Figure 9A). Hydrogen peroxide (H_2_O_2_) contents were measured to confirm the accumulation of ROS in the roots of *phyb* mutant. Subseuently, *phyb* mutants always maintained high H_2_O_2_ contents compared to wild type and in addition showed the highest H_2_O_2_ contents under drought stress (Figure 9B). Measurement of APX enzyme activity between *phyb* mutants and wild type segregants demonstrates that *phyb* mutants have the better performance of APX activity at drought stress for 3 d than that of wild type. In addition, the CAT activity of the *phyb* mutants was consistently higher than that of WT, although it decreased during drought stress rather than normal condition (Figure 9C). Therefore, we estimate that *phyb* mutant might retain the tolerance to drought stress by effective ROS scavenging in root besides controlling stomatal density in leaf.

## Discussion

### RNA-seq analysis using roots of rice plants grown in soil under a limited moisture supply provides novel sources for future applications to enhance drought tolerance

Our candidate genes for conferring tolerance to water deficiencies were identified from RNA-Seq transcriptome analysis of root samples from 4-week-old soil-grown rice plants. The samples were well-qualified for transcriptome analysis based on time-course physiological responses of roots under water deficiency and recovery compared to controls as well as expression patterns analysis with marker genes. Our transcriptome data is useful for identifying novel targets for drought tolerance. When RNA-Seq data were compared with public microarray data under drought stress conditions, 32.05% of those genes (352 of 1,098) showed similar trends in their stress responses (Table S8). However, the others are unique in our experiments, suggesting novel candidate genes for future studies. In addition, the RNA-Seq approach can provide more candidates that cannot be discovered by microarray technology because the latter is able to detect the expression profiles of fixed-target transcripts. In parallel with this description, we did not identify Affymetrix probe IDs for 279 genes (i.e., 25.40% of the total) of DEGs after 3 d of drought treatment. By contrast, the most popular array system in rice, Affymetrix array platform, covers ~90% of the annotated genes in the rice genome (Jung et al., 2008a; Chandran and Jung, 2014). Therefore, unique DEGs under drought treatment in this study might be valuable for future studies and further applications to understand novel regulatory mechanism for drought tolerance responses.

### Drought stress might stimulate degradation of primary metabolites through an ubiquitin-mediated protein degradation pathway or via transcriptional regulation

MapMan analysis can reveal a global view of the metabolic pathways associated with the drought-stress response in roots. Many rice genes in pathways for carbohydrate, cell wall, lipid, and amino acid degradation are stimulated when the water supply is limited, thereby implying that plants use those metabolites and energy resulted from degradation pathways to tolerate stressful growing conditions. For example, a unique Ni^2+^-dependent and methylglyoxal-inducible GLY I functions in the response and adaptation of rice to abiotic stresses (Mustafiz et al., 2014). The phospholipase Dα (PLDα) mediates H(+)-ATPase activity and is involved in salt tolerance (Shen et al., 2011; see also Figure 6A, Table 2). Therefore, we propose that the ubiquitin-mediated pathway for protein degradation associated with our candidate genes is evidence that F-box E3 ligases or RING box E3 ligases have dominant roles in the process. For example, *OsRZFP3*4 (*LOC_Os01g52110*), encoding a rice RING zinc-finger protein, is positively involved in the opening of stomata, even under ABA treatment, and might have a potential role in abiotic-stress tolerance (Hsu et al., 2014). Moreover, the *D3* (*LOC_Os06g06050*) gene, encoding an F-box leucine-rich repeat protein orthologous to *Arabidopsis* MAX2/ORE9, is involved in tillering and shoot-branching (Ishikawa et al., 2005). Our transcriptome data also suggest additional roles in the drought response (Figure 6D, Table 2). Therefore, we believe that many genes within the degradation pathways of primary metabolites, as well as biosynthetic pathways for secondary metabolites, positively influence drought tolerance in rice roots. However, functional elucidation of major, yet uncharacterized, regulatory genes could lead to novel ways for improving the plant response to a water deficiency.

### Combining the protein interactome with network analysis and incorporation of mutants suggests regulatory pathways for tolerating drought stress

In our network analysis, we found that *LOC_Os08g39140*, encoding heat shock protein 90 (HSP90) in circle 71 of the network, potentially interacts with seven gene products. Among them, four have known functions (i.e., Receptor for Activated C-Kinase 1A, or RACK1A; PDCD5, TPS1, and Metal Tolerance Protein 1, or MTP1) while the others are TFs (Figure 7). Heat-shock proteins/chaperones play a crucial role in protecting plants through proteins and membranes stabilization and protein re-folding under stress conditions (Wang et al., 2004). Among the products that interact with HSP90, three are related to abiotic-stress responses, indicating significant roles for HSP90. The three include OsPDCD5, involved in salinity (Yang et al., 2013); OsTPS1, involved in drought, salinity, and cold (Li H. W. et al., 2011); and OsMTP1, which improves plant tolerance to excess heavy metals such as Zn and Cd (Yuan et al., 2012). In addition, RACK1A functions in the production of ROS and resistance to rice blast infection (Nakashima et al., 2008). One inevitable consequence of drought stress is that more ROS are generated in diverse cellular compartments, e.g., the chloroplasts, peroxisomes, and mitochondria. However, ROS accumulations in response to a water deficiency are tightly controlled by a versatile and cooperative antioxidant system that modulates intracellular ROS concentrations and sets the redox-status of a cell (Cruz de Carvalho, 2008). These activities suggest that RACK1A is involved in a ROS-mediating drought-stress pathway.

The three other TFs with important roles in drought tolerance include, first, a zinc finger domain (circle 11 in our network). Overexpression of ZFP36 in rice elevates the activities of antioxidant enzymes and enhances plant tolerance to water and oxidative stresses (Zhang et al., 2014). Second, a loss-of-function mutant of a drought and salt tolerance gene (*DST*) stimulates stomatal closure and reduce stomatal density, resulting in greater drought and salt tolerance in rice (Huang et al., 2009). Finally, an upstream activation factor (UAF in circle 16) regulates the pace of transcriptional activity by binding to an upstream element of target genes in response to diverse signals (Carthew et al., 1985). Therefore, our network data that integrate this diverse functional information will be useful resources when designing more detailed molecular mechanisms for future applications that can enhance tolerance to a water deficiency.

### A model of drought tolerance mechanism by modulating reactive oxygen species during root development

Hydrogen peroxide (H_2_O_2_) is a signaling molecule involved in the regulation of specific biological and physiological processes and is a relatively stable non-radical ROS. However, abiotic stresses such as drought increase the production of H_2_O_2_ in plants, which can cause serious damage to biomolecules (Slesak et al., 2007). To solve this problem, plants are endowed with H_2_O_2_-metabolizing enzymes, among which CAT and APX play the most important role (Sofo et al., 2015; Li et al., 2016). For example, *Panicum sumatrense* with strong drought tolerance has been reported to increase the activity of antioxidant enzymes such as CAT and peroxidase during drought stress (Ajithkumar and Panneerselvam, 2014). Olive plants also increase CAT activity in the leaves when water deficit conditions, thereby limiting cellular damage due to ROS (Sofo et al., 2004). As the drought increased in soybeans, a clear increase in the amount and activity of APX was observed and APX2 overexpressing plants in rice showed increased APX activity and improved stress resistance (Kausar et al., 2012; Zhang et al., 2013). These facts support that CAT and APX are antioxidant enzymes that play a key role in recovery from drought stress (Faize et al., 2011; Pinheiro and Chaves, 2011). We confirmed an increase in APX activity in the *phyb* mutant roots during drought stress. In addition, we observed that *phyb* mutant had higher CAT activity than WT under any circumstances (Figure 9C).

Despite the higher level of APX and CAT activity, *phyb* mutants have higher H_2_O_2_ accumulation than wild type plants. One possible explanation on this phenamenum is that rice plants under drought stress might use H_2_O_2_ as a second messenger to trigger ROS removing pathway. It was demonstrated that H_2_O_2_ plays key roles in processing external biotic and abiotic stimuli thorugh signal transduction pathways in plant cells (Petrov and Van Breusegem, 2012). In addition, several reports in rice support the significance of increased H_2_O_2_ in tolerance responses against drought stress: overexpression of *abscisic acid, stress, and ripening* 5 (*OsASR5*) gene exhibits a drought resistant phenotype despite higher H_2_O_2_ content under drought stress compared to wild type (Li J. et al., 2011); *drought and salt tolerance* (*dst*) mutant has increased stomatal closure and reduced stomatal density due to accumulated H_2_O_2_, consequently resulting in enhanced drought tolerance in rice (Huang et al., 2009); rice *similar to RCD one* (*SRO/OsSRO1c*) promotes stomatal closure and H_2_O_2_ accumulation via stress-responsive NAC 1 (SNAC1) and DST regulators, resulting in tolerance responses against drought stress (You et al., 2013).

**Figure 10:**
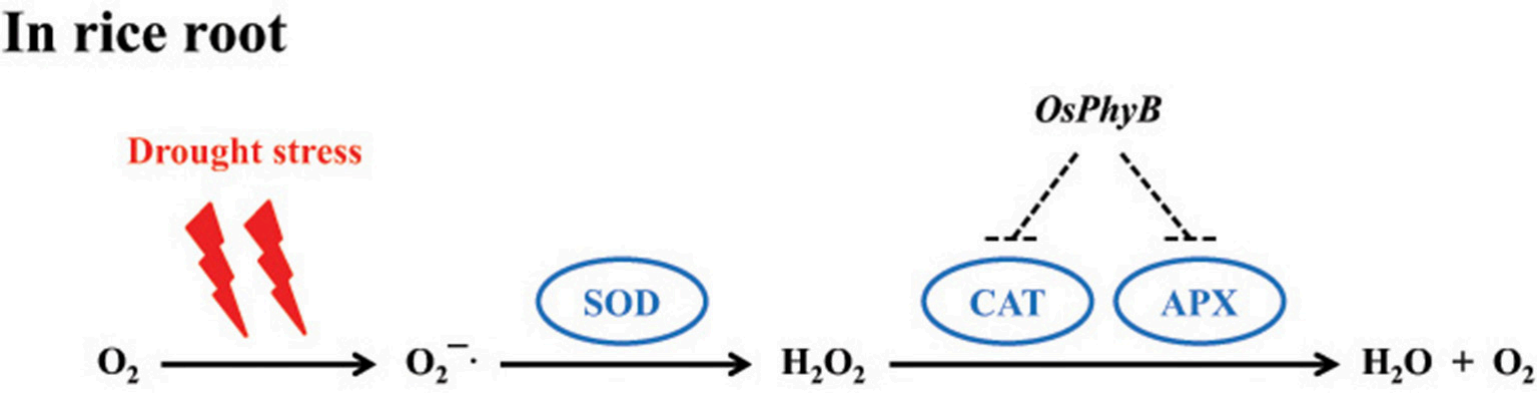
**Scavenging model of reactive oxygen species (ROS) in rice roots under drought stresses**. APX and CAT, which play a key role in ROS scavenging, are thought to be regulated by *phyB*.

Recent studies indicate that phytochrome influences the growth and development of roots through the role of photoreceptors and their associated signaling mechanisms. For example, in *Arabidopsis*, light was efficiently conducted through the stems to the roots, resulting in the accumulation of ELOTATED HYPOCOTYL 5 (HY5) protein by photoactivated *phyb* (Lee et al., 2016). We therefore expect that *OsPhyB* acts as a negative regulator of APX and CAT activity by a transcription factor or phytochrome interacting factor (PIF) during drought stress in rice roots (Figure 10). Details of this model need to be elucidated through further studies.

## Author contributions

YY, JP, and YG performed the experiments; YY and AN analyzed the data; and YY, SL, GA, and KJ wrote the paper. All authors read and approved the final manuscript.

### Conflict of interest statement

The authors declare that the research was conducted in the absence of any commercial or financial relationships that could be construed as a potential conflict of interest.

## Supplementary material

Table S1 **Primer sequences used for genotype and qRT-PCR analyses**.

Table S2 **Names and related RNA-Seq data for genes differentially expressed in response to 2 d of water deficiency (WD) vs. well-watered control (C)**.

Table S3 **Names and related RNA-Seq data for genes differentially expressed in response to 3 d of water deficiency (WD) vs. well-watered control (C)**.

Table S4 **Gene Ontology (GO) assignment in “biological process” category for genes up-regulated by 3 d of WD treatment**.

Table S5 **Overview of MapMan terms assigned to genes up-regulated by 3 d of WD treatment**.

Table S6 **Detailed information about MapMan terms assigned to genes up-regulated by 3 d of WD treatment**.

Table S7 **Detailed information from analyses of protein–protein interactions for drought-stress response in Figure 7**.

Table S8 **Comparison of microarray data (GSE26280) and RNA-Seq data for roots from drought-stressed rice plants**.

Figure S1 **Number of upregulated and downregulated genes identified from comparisons between roots of plants exposed for 2 or 3 d to WD treatment and corresponding well-watered controls**.

Figure S2 **Validation of two water deficiency-induced genes** [**(A)**; *LOC_Os04g52290* and **(B)**; *LOC_Os07g02710*] revealed by promoter trap system and *GUS* assays.
