# Supplementary figures and images for: OsPhyB-Mediating Novel Regulatory Pathway for Drought Tolerance in Rice Root Identified by a Global RNA-Seq Transcriptome Analysis of Rice Genes in Response to Water Deficiencies
Source: Front Plant Sci. 2017 Apr 26;8:580. doi: 10.3389/fpls.2017.00580 (PMC5405136; doi:10.3389/fpls.2017.00580)

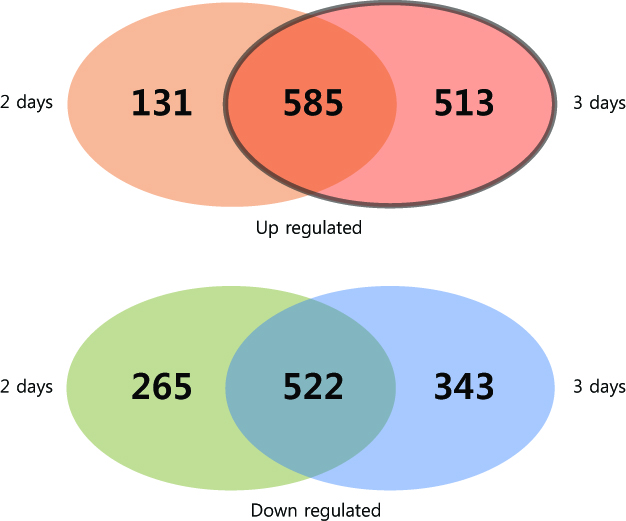

Supplement: Figure S1 — Number of upregulated and downregulated genes identified from comparisons between roots of plants exposed for 2 or 3 d to WD treatment and corresponding well-watered controls. [file Image1.JPEG]

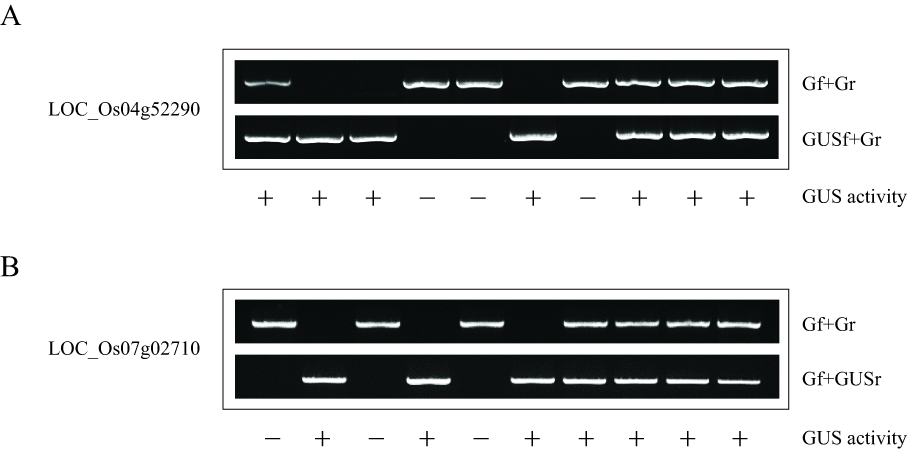

Supplement: Figure S2 — Validation of two water deficiency-induced genes [(A); LOC_Os04g52290 and (B); LOC_Os07g02710] revealed by promoter trap system and GUS assays. [file Image2.JPEG]
